# Mesenchymal stem cell response to topographically modified CoCrMo

**DOI:** 10.1002/jbm.a.35514

**Published:** 2015-06-19

**Authors:** Niall Logan, Laurent Bozec, Alison Traynor, Peter Brett

**Affiliations:** ^1^ Biomaterials and Tissue EngineeringUniversity College London, Eastman Dental InstituteLondonWC1X 8LDUnited Kingdom; ^2^ Corin LtdCirencester, GloucestershireGl7 1YJUnited Kingdom

**Keywords:** mesenchymal stem cells, osteogenic differentiation, adhesion, SLA, cobalt alloy

## Abstract

Surface roughness on implant materials has been shown to be highly influential on the behavior of osteogenic cells. Four surface topographies were engineered on cobalt chromium molybdenum (CoCrMo) in order to examine this influence on human mesenchymal stem cells (MSC). These treatments were smooth polished (SMO), acid etched (AE) using HCl 7.4% and H_2_SO_4_ 76% followed by HNO_3_ 30%, sand blasted, and acid etched using either 50 μm Al_2_O_3_ (SLA50) or 250 μm Al_2_O_3_ grit (SLA250). Characterization of the surfaces included energy dispersive X‐ray analysis (EDX), contact angle, and surface roughness analysis. Human MSCs were cultured onto the four CoCrMo substrates and markers of cell attachment, retention, proliferation, cytotoxicity, and osteogenic differentiation were studied. Residual aluminum was observed on both SLA surfaces although this appeared to be more widely spread on SLA50, whilst SLA250 was shown to have the roughest topography with an *R*_a_ value greater than 1 μm. All substrates were shown to be largely non‐cytotoxic although both SLA surfaces were shown to reduce cell attachment, whilst SLA50 also delayed cell proliferation. In contrast, SLA250 stimulated a good rate of proliferation resulting in the largest cell population by day 21. In addition, SLA250 stimulated enhanced cell retention, calcium deposition, and hydroxyapatite formation compared to SMO (*p* < 0.05). The enhanced response stimulated by SLA250 surface modification may prove advantageous for increasing the bioactivity of implants formed of CoCrMo. © 2015 Wiley Periodicals, Inc. J Biomed Mater Res Part A: 103A: 3747–3756, 2015.

## INTRODUCTION

The average life expectancy in the UK has risen 4.2 years between 1990 and 2010.^1^ One of the many consequences of this situation is an increasing demand for arthroplasty procedures.^2^ This growing demand for total hip replacements (THR) and total knee replacements (TKR) is not restricted solely to the UK, but is an international trend,^3^ with the USA predicted to see a drastic increase in arthroplasty procedures by the year 2030.^4^, ^5^ As a result of this, what was once deemed to be acceptable as sufficient implant performance in orthopedic devices is no longer so, as patients now require devices that promote reduced recovery times, alongside increased longevity.

To increase the performance of a biomaterial, the biological response at the bone implant interface can be influenced by surface modification of the implant.^6^ In modifying the implant surface, cell behavior can be controlled and manipulated to promote a desired response.^7^ Methods of surface modification are highly varied, ranging from micron scale machining and etching,^8^ to the formation of grooves,^9^ tubes,^10^ pores,^11^ and pillars^12^, ^13^ on the nanoscale.

One such method of topographical surface modification used widely on titanium is sand blasting followed by acid etching (SLA), which has been shown to be capable of promoting a desirable response *in vitro* and *in vivo*.^14^, ^15^ The dual step procedure is thought to form a microscale topography through the blasting process, which is then followed by the addition of a nanoscale topography via acid etching.^15^

>Despite the general acceptance that the material of choice for orthopedic applications is titanium and its alloys, other more mechanically strong materials are often preferred in some procedures.^16^ Cobalt chromium molybdenum (CoCrMo) lacks the bioactivity of titanium, but is widely used in orthopedic applications where a mechanically superior material is required. Efforts to improve the bioactivity of CoCrMo have included chemical vapor deposition coating,^17^ sol–gel coating,^18^ ultraviolet photofunctionalization,^19^, ^20^ and immobilization with bone morphogenic peptides.^21^, ^22^, ^23^ Surface modification of CoCrMo through topographical methods remains largely un‐investigated.

The present study sought to investigate whether the SLA surface modification used widely on titanium could be replicated onto CoCrMo in an attempt to increase the bioactivity of the material. Samples of CoCrMo were prepared as smooth, acid etched, SLA using 50‐μm Al_2_O_3_ grit and SLA using 250‐μm Al_2_O_3_ grit, and characterized using SEM, contact angle, and surface roughness analysis. Human mesenchymal stem cells (MSCs) were utilized to study the changes in bioactivity induced by all four surface topographies by measuring cell proliferation, attachment, retention, cytotoxicity, and osteogenic differentiation in the form of calcium deposition, hydroxyapatite formation, and alkaline phosphatase (ALP) activity.

## MATERIALS AND METHODS

### Sample preparation

Discs formed of CoCrMo (Cr 26–30%, Mo 5–7%), dimensions of Ø 15 mm, 1 mm thickness, were supplied with a machined finish which was removed as previously described to create a smooth (SMO) topography.^17^, ^19^ SMO discs were then used to engineer three other topographies; acid etched (AE), sand blasted acid etched using 50 µm Al_2_O_3_ grit (SLA50), and sand blasted acid etched using 250 µm Al_2_O_3_ (SLA250). AE substrates were created by following a previously reported protocol,^24^ which involved acid etching in HCl 7.4% and H_2_SO_4_ 76% for 6 min at 100°C, followed by HNO_3_ 30% for 5 min at 60°C. All samples were rinsed under H_2_O between etches and finally sonicated in isopropanol (15 min at 30°C) followed by _dd_H_2_O (10 min at room temperature (RT)) and air dried. SLA50 and SLA250 substrates were created by fixing SMO discs on a mount and subsequently grit blasting at 95 Psi at a distance of 5 cm (Vaniman, Sandstorm 2, 80301). 250 µm Al_2_O_3_ was used as the blasting media for SLA250 substrates (Renfert, Cobra, 15851005), whilst 50 µm Al_2_O_3_ media was used for SLA50 (Renfert, Cobra, 15941205). SLA50 and SLA250 substrates were then sonicated in isopropanol (15 min at 30°C) followed by _dd_H_2_O (10 min at RT) before undergoing the same acid etching protocol as AE. Before the discs were used for cell culture experiments all discs were washed thoroughly in _dd_H_2_O, air dried, and sterilized by ultraviolet light irradiation for 20 min on each side (BONMAY, BR‐506).

### Characterization

Characterization of SMO, AE, SLA50, and SLA250 substrates was performed by studying surface wettability, roughness, and elemental analysis. Contact angle measurements were taken using an optical contact angle meter (KSV Instruments, Cam 200) with drops of _dd_H_2_O (*n* = 10). The surface topography of each substrate was analyzed by laser profilometry (Scantron, Proscan 100) where a total area of 6.25 mm^2^ was examined on each surface (*n* = 3). Roughness values in the form of *R*_a_ were then calculated using Proscan software. In addition, scanning electron microscopy (SEM) combined with energy dispersive X‐ray analysis (EDX) was performed to examine the surface chemistries of the four surfaces.

### Cell culture

Human MSCs isolated from the bone marrow of three donors were acquired from the Institute for Regenerative Medicine, Texas A & M Health Science Center College of Medicine. Expression of stem cell markers, as well as adipogenic, chondrogenic, and osteogenic differentiation markers had been assessed during pre‐characterization studies. Cells were seeded on tissue culture plastic at a density of 740 cells per cm^2^ and expanded in α minimum essential medium (MEM, Gibco, 22571‐020) containing 10% fetal bovine serum (FBS, Invitrogen, 10270106) and 1% penicillin/streptomycin (PS, Sigma‐Aldrich, P0781). Cells were then incubated at standard culture conditions of 37°C, 5% CO_2_ in a humidified atmosphere until they reached 80% confluence, when they were then harvested by trypsin (0.05%/EDTA (0.002%) (Life technologies, R‐001‐100). When osteogenic media (OM) was employed it consisted of Dulbecco's Modified Eagle's Medium (DMEM, Gibco, 31885‐023) containing 10% FBS, 1% PS, and further supplemented with β‐glycerol phosphate (Sigma‐Aldrich, G9891), l‐ascorbic acid (Sigma Aldrich, A8960), and dexamethasone (Sigma‐Aldrich, D9402). To ensure integrity of the results, MSCs of low passage were used for the experiments (<5).

### Live/Dead

MSCs were seeded at 3.5 × 10^4^ cells per well onto SMO, AE, SLA50, and SLA250 substrates in a 24‐well plate (*n* = 3). After 24‐h incubation at standard culture conditions, MSCs were washed twice in 1 mL of Dulbecco's phosphate buffered saline (PBS, Lonza, 17‐512F) and incubated with Live/Dead assay reagent for 15 min in the dark at RT (Life technologies, R37601). Following this, substrates were viewed using a fluorescence microscope fitted with the appropriate filters (Leica, DMIRB).

### Proliferation

MSCs from three donors were seeded at an initial density of 2 × 10^3^ cells per well on SMO, AE, SLA50, and SLA250 substrates (*n* = 3). Individual plates were setup for growth media (GM) and OM. Both plates were incubated at standard culture conditions and had the media replaced at each time point of analysis. The number of cells at each time point was measured by the AlamarBlue assay (AbD Sertoec, BUF012B). 100 μL of assay reagent was added to each well containing 1 mL of media and cells were incubated for 4 h at standard culture conditions. Following this, two 100 μL aliquots of supernatant were removed from each well for analysis using a fluorescence plate reader (BioTek, FLX800, Excitation *λ* = 530 nm, emission *λ* = 590 nm). Cell numbers were calculated through interpolation via a standard curve.

### Attachment

Cell attachment was studied using MSCs from three donors seeded at density 4 × 10^4^ cells per well on SMO, AE, SLA50, and SLA250 substrates (*n* = 3). Following 24‐h incubation at standard culture conditions in GM, the media in each well was removed and replaced, and the remaining cells were quantified using AlamarBlue as previously described.

### Retention

To gain an understanding of how well the remaining cells were attached to the substrate surface, a retention study was performed. MSCs from three donors were seeded at 3.5 × 10^4^ cells per well on SMO, AE, SLA50, and SLA250 substrates in GM (*n* = 3). Cells were incubated for 24 h at standard culture conditions and then underwent three thorough washes using PBS on an orbital shaker (60 s at 60 rpm). The remaining cells were then quantified using AlamarBlue as previously described.

### Osteogenic assays

Markers of both the early and late stages of the osteogenic differentiation process were studied. ALP activity was studied after 5 days in culture, whilst calcium deposition and hydroxyapatite formation was analyzed following 21 days in culture.

For ALP, MSCs from three donors were seeded at 2 × 10^4^ cells per well in OM on SMO, AE, SLA50, and SLA250 substrates (*n* = 3). Prior to performing the ALP assay the number of cells was quantified using AlamarBlue as previously described. A colorimetric assay was then used to assess ALP activity as per the manufacturer's instructions (Anaspec, SensoLyte AS‐72146). Cells were washed twice with assay buffer then homogenized using triton X‐100, before being transferred to a micro‐centrifuge tube and stored at 4°C for 10 min. The cell suspension was then centrifuged at 2500*g* for 10 min at 4°C to form a pellet. About 50 µL of each sample was then combined with 50 µL *p*‐nitrophenyl phosphate substrate solution in a 96‐well plate and left for 60 min in the dark at RT. Following this, the optical density was measured at 405 nm (Tecan, M200) and concentrations were calculated through use of concentration standards.

The concentration of calcium ions was measured using a colorimetric assay as per the manufacturer's instructions (Bio Assay Systems, QuantiChrom, DICA‐500). MSCs from three donors were seeded at 12.5 × 10^3^ cells per well in OM on SMO, AE, SLA50, and SLA250 (*n* = 3). AlamarBlue was used to measure the number of cells prior to performing the assay. Cells were washed twice using PBS before being incubated with 500 µL 1*M* HCl for 60 min at RT on a rocking plate. A 5 µL aliquot from each sample was then transferred to a 96‐well plate where it was combined with 200 µL assay reagent, and calcium levels were obtained (*λ* = 612 nm, Tecan, M200) with concentrations calculated through use of known concentration standards.

Hydroxyapatite was analyzed by both fluorescence microscopy and fluorescent plate reader using the OsteoImage mineralization assay (Lonza, PA‐1503). MSCs from three donors were seeded at 12.5 × 10^3^ cells per well in OM on SMO, AE, SLA50, and SLA250 substrates (*n* = 3). The cells were washed twice using PBS and fixed for 15 min by 4% paraformaldehyde. After fixation, cells were washed a further two times using wash buffer and then incubated with staining reagent for 30 min at RT in the dark. After three additional washes, the amount of hydroxyapatite was measured using a fluorescent plate reader (Excitation *λ* = 492 nm, emission *λ* = 520 nm, BioTek, FLX800). Visual detection of hydroxyapatite was also performed using a fluorescence microscope fitted with the appropriate filters (Leica, DMIRB).

### Statistical analysis

Human MSCs from three donors (*N* = 3) were used in triplicate (*n* = 3) throughout the study. SEM EDX scans were performed at *n* = 8, contact angle analysis was performed at *n* = 10 and roughness scans were completed at *n* = 3. Statistical analysis was carried out using the students *t* test in GraphPad Prism software (v5.04) with *p* < 0.05 deemed to be statistically significant.

## RESULTS

### Contact angle

**Figure 1:**
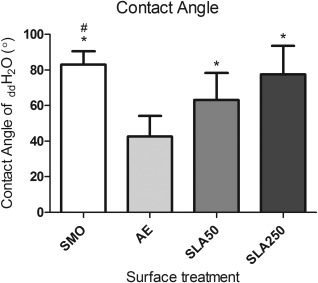
Contact angle of _dd_H_2_O on each substrate. Each column represents the mean ± 1 standard deviation (SD) (*n* = 10). *p < 0.05 substrate verses AE, # *p* < 0.05 substrate verses SLA50.

As shown in Figure 1, the most hydrophobic of the four topographies was SMO (83.11° ± 7.41°). AE was shown to be the most hydrophilic of the four (42.70° ± 11.45°), with a lower contact angle than both SLA50 (63.09° ± 15.26°) and lastly SLA250 (77.60° ± 16.05°).

### Roughness

**Figure 2:**
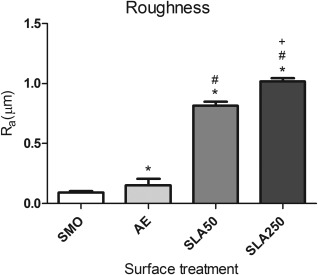
Surface roughness values of all four topographies. Each bar represents the mean ± 1 SD (*n* = 3). *p < 0.05 substrate verses SMO, # *p* < 0.05 substrate verses AE, + *p* < 0.05 substrate verse SLA50.

The roughness of each substrate was analyzed by laser profilometry with the results shown in Figure 2. The SMO substrate (0.09 ± 0.01) was found to have the lowest *R*_a_ value which was significantly less rough compared to AE (0.15 ± 0.05) (*p* < 0.05). Both SLA50 (0.82 ± 0.03) and SLA250 (1.02 ± 0.03) were significantly rougher than SMO and AE, whilst SLA250 was also found to have a significantly greater *R*_a_ value compared to SLA50 (*p* < 0.05).

### SEM and EDX

**Figure 3:**
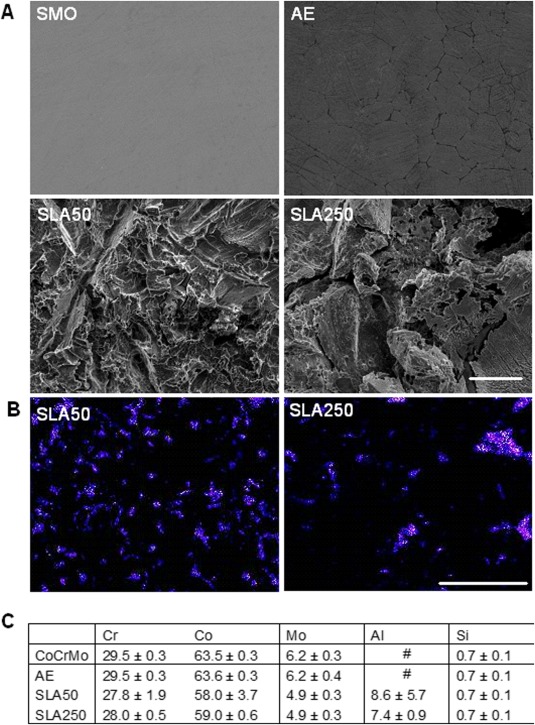
A: SEM images displaying surface topography of the four substrates. Scale bar = 10 μm. B: EDX mapping of aluminum on SLA50 and SLA250, showing variation in residual Al_2_O_3_ particle distribution on the substrate surface. Scale bar = 100 μm. C: Quantitative % data from EDX scans (*n* = 8). [Color figure can be viewed in the online issue, which is available at wileyonlinelibrary.com.]

SEM analysis showed an almost featureless topography on SMO and the formation of what appeared to be grain boundaries on AE [Fig. 3(A)]. SLA substrates were evidently different from SMO and AE, with the presence of peaks and pits visible on the substrate surface [Fig. 3(A)]. EDX was utilized to detect the presence of residual alumina on the substrate surface following surface modification techniques. As expected, no aluminum was found on either SMO or AE substrates as they did not undergo sandblasting. Residual aluminum was observed on both SLA50 (8.60 ± 5.71) and SLA250 (7.37 ± 0.97) substrates [Fig. 3(C)]. A larger amount of aluminum was found on the SLA50 substrate although this was not statistically significant. Interestingly, the distribution of residual aluminum on the substrate surface was noticeably different between SLA50 and SLA250. As shown in Figure 3(B), the SLA50 substrate appeared to have a greater spread of aluminum in the form of numerous small patches. This was not found to the same extent on SLA250, which appeared to have fewer residual particles that were noticeably larger in size.

### Live/Dead

**Figure 4:**
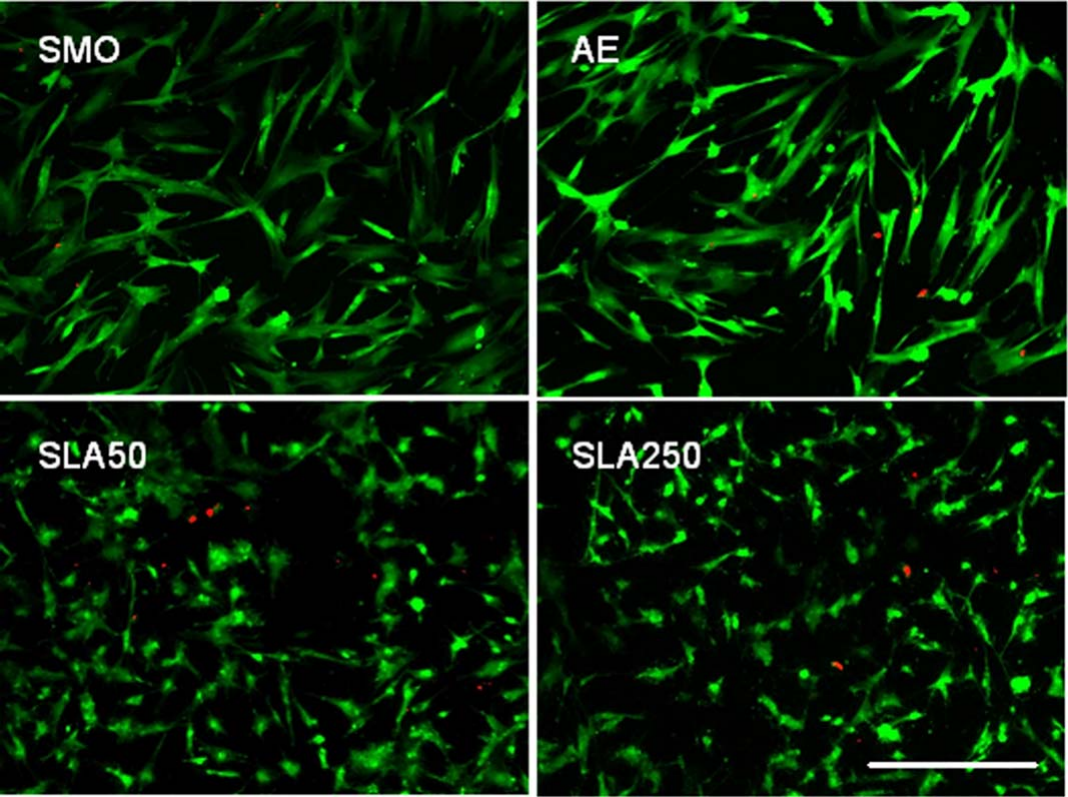
Live/Dead images displaying human MSCS on all four topographies. Green: live cells stained with calcein AM. Red: dead cells. Scale bar = 100 μm. [Color figure can be viewed in the online issue, which is available at wileyonlinelibrary.com.]

Live/Dead staining was used to ascertain the cytotoxicity of the four surface topographies. Using fluorescence microscopy, all four substrates showed acceptable cytocompatibility (Fig. 4). A number of dead cells that were positive for the red fluorescent marker were observed on each substrate, although they appeared to be most frequent on SLA50.

### Proliferation

**Figure 5:**
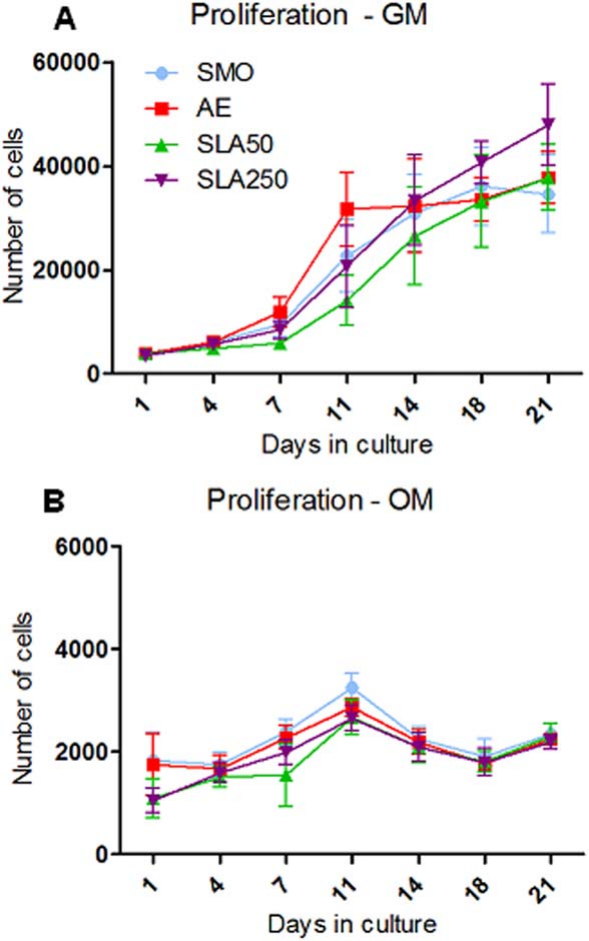
Proliferation data of human MSCs in GM (A) and OM (B). Each point depicts the mean ± 1 SD (*N* = 3) (*n* = 3). [Color figure can be viewed in the online issue, which is available at wileyonlinelibrary.com.]

The rate of proliferation in both OM and GM was studied for 21 days as shown in Figure 5. In GM the AE substrate stimulated the fastest rate of proliferation up to day 11, whilst SLA50 appeared to delay cellular proliferation. SLA250 was capable of promoting proliferation beyond day 14 when the other substrates seemed to reach a level of confluence, and consequently, SLA250 had the largest cell population by day 21. Proliferation in OM was similar across all substrates although a smaller number of cells were found on both SLA surfaces after 24 h compared to SMO and AE. There was a distinct difference in the rate of proliferation observed in cells cultured in GM [Fig. 5(A)] and OM [Fig. 5(B)]. GM stimulated a rapid rate of cell growth whilst the use of OM appeared to slow down and inhibit cellular proliferation.

### Attachment

**Figure 6:**
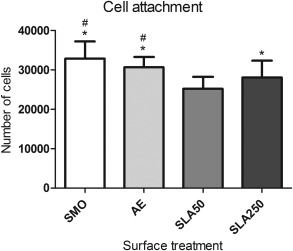
Cell attachment data following 24 h incubation. Each bar represents the mean ± 1 SD (*N* = 3) (*n* = 3). *p < 0.05 substrate verses SLA50. #*p* < 0.05 substrate verses SLA250.

Cell attachment was studied after 24 h in culture. Both SMO (32,815 ± 4380) and AE (30,635 ± 2650) had significantly more attached cells than the SLA50 (25,123 ± 3015) and SLA250 (28,017 ± 4305) substrates as seen in Figure 6 (*p* < 0.05). In addition, SLA250 had significantly more attached cells compared to SLA50 (*p* < 0.05).

### Retention

**Figure 7:**
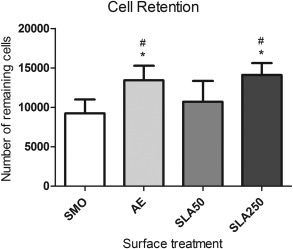
Cell retention data displaying the remaining cell population following three mechanical washes. Each bar represents the mean ± 1 SD (*N* = 3) (*n* = 3). *p < 0.05 substrate verses SMO. #*p* < 0.05 substrate verses SLA50.

To gain an understanding of how well adhered the MSCs were to the substrate surface, cell retention was studied after three mechanical washes in PBS following 24 h in culture. As shown in Figure 7, both AE (13,431 ± 1827) and SLA250 (14,105 ± 1479) substrates had significantly more remaining cells compared to SMO (9221 ± 1765) and SLA50 (10,709 ± 2652) (*p* < 0.05). This data implies that those cells attached to AE and SLA250 are better adhered to the substrate surface than those found on SMO and SLA50.

### Calcium deposition

**Figure 8:**
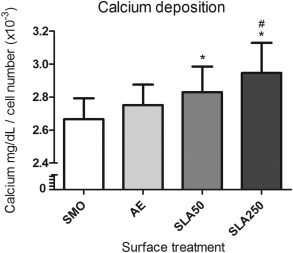
Calcium deposition per cell after 21 days culture in OM. Each bar represents the mean ± 1 SD (*N* = 3) (*n* = 3). *p < 0.05 substrate verses SMO. #*p* < 0.05 substrate verses AE.

The level of osteogenic differentiation occurring on the surface of a substrate can be associated with the rate of mineralization. To monitor this process, the amount of calcium per cell was quantified on each substrate after 21 days culture in OM. As shown in Figure 8, both SLA surfaces stimulated significantly greater calcium deposition than that found on SMO (*p* < 0.05). SLA250 was also found to have significantly more calcium per cell than AE (*p* < 0.05), which indicates that MSCs were differentiating at a superior rate on SLA250.

### Hydroxyapatite formation

**Figure 9:**
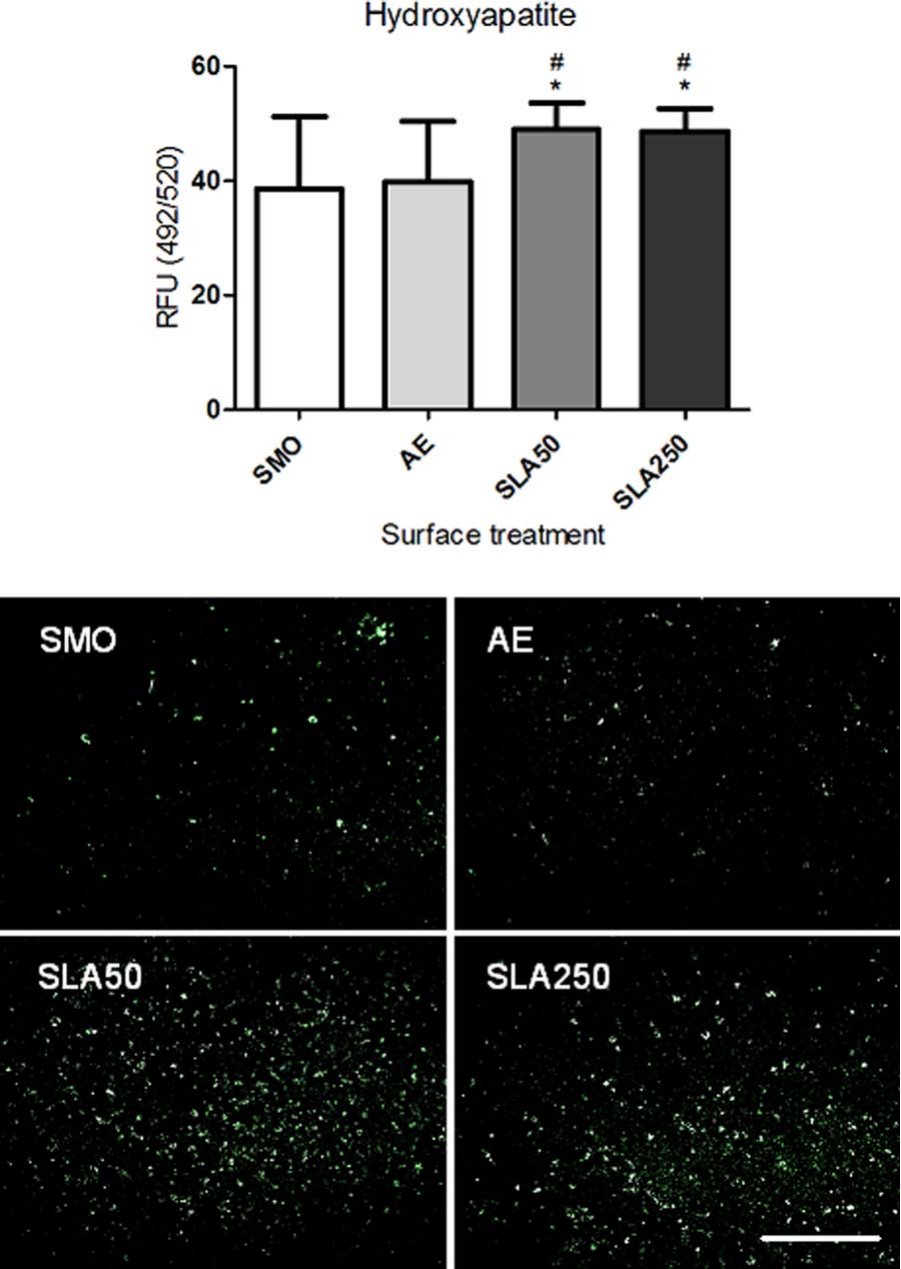
Above: Fluorescent plate reader data showing hydroxyapatite formation after 21 days in culture. Each bar represents the mean ± 1 SD (*N* = 3) (*n* = 3). *p < 0.05 substrate verses SMO. #*p* < 0.05 substrate verses AE. Below: Fluorescence microscope images showing hydroxyapatite distribution on the substrate surface. Green depicts hydroxyapatite nodules. Scale bar = 800 µm. [Color figure can be viewed in the online issue, which is available at wileyonlinelibrary.com.]

Hydroxyapatite formation is widely recognized as a late marker of mineralization and osteogenic differentiation. After 21 days in culture, hydroxyapatite formation was quantified by fluorescent plate reader and visually assessed via fluorescence microscopy. As shown in Figure 9, there was a significantly stronger hydroxyapatite signal present on both SLA50 and SLA250 compared to SMO and AE (*p* < 0.05). Microscopy analysis confirmed the widespread deposition of hydroxyapatite on the surface of SLA50 and SLA250 substrates, implying that a greater level of mineralization was occurring on these substrates compared to SMO and AE.

### ALP

The level of ALP activity per cell was studied after 5 days in culture in OM. No significant difference was observed in cells across the four substrates.

## DISCUSSION

The mechanical strength of CoCrMo and its excellent resistance to wear make it a desirable material for use in orthopedic applications, where devices must be long‐lasting and capable of withstanding the harsh cyclic loading of the joint. Despite CoCrMo's superior mechanical properties, it lacks the biocompatibility of other bio‐metals such as titanium.^16^ The present study aimed to investigate whether the bioactivity of CoCrMo could be enhanced through topographically modifying the surface of the material in an attempt to create an environment that enhanced osteoinduction and osteoconduction in human MSCs.

MSCs can be affected by topographical features present on a material surface.^25^ To prevent this occurring, all CoCrMo discs were initially ground and polished to a smooth featureless finish as previously described,^17^ with discs at this stage used as a smooth control. Three additional topographies were then created via a combination of sandblasting and acid etching techniques.

Surface roughness of the four substrates was analyzed by laser profilometry. As shown in Figure 2, it was found that SMO had the lowest *R*_a_ value. AE had the second lowest *R*_a_ value which was followed by SLA50, leaving SLA250 as the most rough substrate, with an *R*_a_ value of 1.02 ± 0.03 μm. Moderately rough surfaces with a *R*_a_ value within range of 1–2 μm are suggested to promote the strongest bone response compared against smooth (*R*_a_ 0–0.5 µm), minimally rough (*R*_a_ 0.5–1 µm), and rough surfaces (*R*_a_ > 2 µm).^26^ This data implies that the rough topography formed by sandblasting with 250 μm Al_2_O_3_ grit may be best suited at enhancing mineralization.

Contact angle measurements using _dd_H_2_O were used to study the wettability of the substrates. Interestingly, those substrates that had undergone the acid etching process appeared to have different surface properties to the SMO substrates (Fig. 1). AE had the lowest contact angle of the four substrates, with both SLA50 and SLA250 showing an increase in contact angle that correlated to surface roughness. In contrast, SMO did not follow this trend, as it had the lowest *R*_a_ value, yet was observed to be the most hydrophobic. Nitric acid was recently reported to be an effective method of activating the surface of CoCrMo.^27^ It was shown that treatment with nitric acid increased the wettability of CoCrMo, due to the removal of surface contaminants and introduction of hydrophilic groups at the material surface. Surface contaminants such as hydrocarbons can reduce osteoblast activity.^28^ This indicates that acid etching may not only be advantageous by way of modifying the substrate topographically, but also chemically through surface cleansing.

Elemental analysis of each substrate was performed using EDX. Despite the implementation of thorough cleaning steps throughout sample production, the presence of residual aluminum particles was present on SLA surfaces, which has been reported previously on other engineered SLA surfaces.^29^ SLA50 had a larger amount of residual aluminum, although this difference was not significant (*p* = 0.4748). Interestingly, visual analysis of the location of residual aluminum was noticeably different between SLA50 and SLA250. As shown in Figure 3, SLA50 appeared to have a greater amount of residual particles that were comprehensively spread across the material surface. In contrast, aluminum on SLA250 was congregated in specific areas and as a result, had large sections of the surface that were not contaminated with aluminum.

The cytocompatibility of the substrates was analyzed by Live/Dead staining after 24 h in culture. As shown in Figure 4, all substrates were largely non‐toxic, although of the four, SLA50 appeared to have the largest number of dead cells. An increased number of apoptotic cells after 24 h was also reported on SLA titanium compared to smooth^30^ which implies that there may be a topographical property of the SLA surface which triggers some apoptosis in human MSCs.

The ability of a bio‐metal to promote cellular proliferation is important factor in regards to implant performance. It was observed that in GM, the AE substrate promoted an accelerated rate of proliferation [Fig. 5(A)] which has been previously reported on acid‐etched titanium substrates.^31^ In addition, the lower contact angle found on AE may have affected the rate of proliferation, as material wettability has been shown to be influential in this process, with hydrophilic surfaces reported as superior over their hydrophobic counterparts.^20^, ^32^ It was also found that SLA50 delayed MSC proliferation at early time points, which may be a result of aluminum contamination, as previously reported.^33^ However despite having the similar amounts of aluminum residue, SLA250 did not inhibit proliferation and promoted adequate cell growth to obtain the largest cell population of the four surfaces by day 21. This difference might be related to the distribution of the residues seen between SLA50 and SLA250 (Fig. 3). Interestingly, a somewhat lower number of cells were observed on both SLA surfaces after 24 h in GM and OM [Fig. 5(A,B)] although by day 4, a comparable number were found on all substrates. The variation observed in the rate of cellular proliferation in GM and OM can be accounted to the presence of osteogenic supplements in the OM. These supplements trigger the MSCs to differentiate along their osteogenic linage to form osteoblasts and as a consequence can reduce proliferation.^19^, ^34^

Cell attachment was analyzed after 24 h in GM. It was found that significantly more cells had attached to SMO and AE compared to SLA50 and SLA250 (Fig. 6). In addition, SLA250 was found to have significantly more attached cells compared to SLA50. This data correlates with the reduction in the number of cells observed after 24 h during the proliferation experiment [Fig. 5(A,B)]. It is likely that instead of inhibiting early stage cellular proliferation, the SLA substrates are stimulating a degree of apoptosis, resulting in a reduction in cell attachment, a phenomenon also seen on roughened titanium.^30^ Figure 4 shows a higher number of dead cells on SLA50 which supports this interpretation of the data.

In addition to cell attachment, a measure of how well MSCs were adhered to the substrate surface was analyzed by counting the remaining cells following three mechanical washes in PBS. Both AE and SLA250 substrates had significantly more remaining cells compared to SMO and SLA50 after the mechanical washes (Fig. 7). The lower contact angle found on AE may have contributed to a higher number of adherent cells, as increased hydrophilicity has been shown to enhance cell adhesion by way of increased actin development and focal adhesion formation.^35^ In addition to wettability, increases in cell adhesion have been reported on sand blasted titanium substrates with increased roughness,^31^ implying that the roughness formed on SLA250 may be advantageous for MSC adhesion.

**Figure 10:**
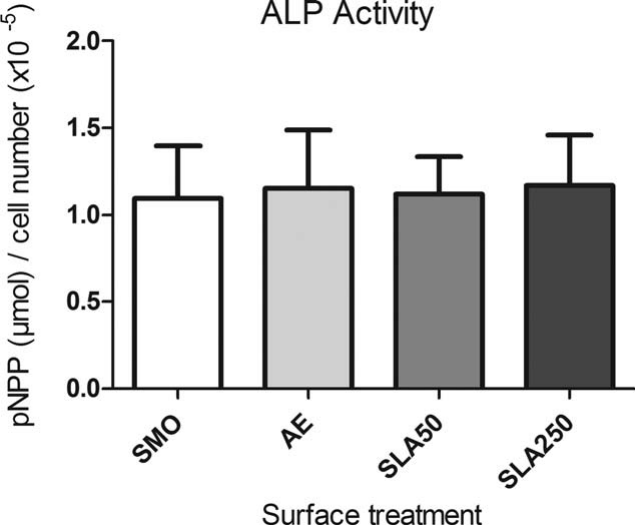
ALP activity per cell after 5 days culture in OM. Each bar represents the mean ± 1 SD (*N* = 3) (*n* = 3).

Another important factor for implant performance is the rate at which the device can promote the osteogenic differentiation of MSCs. MSCs are the first cells with osteogenic potential recruited to the site of implant placement, where they differentiate into osteoblasts capable of forming new bone tissue.^36^, ^37^ If the implant surface can be modified to enhance this process, it should result in increased implant stability and osseointegration.^38^ In the present study it was found that both SLA substrates promoted a significantly higher level of osteogenic differentiation compared to SMO and AE, through the deposition of calcium (Fig. 8) and formation of hydroxyapatite (Fig. 9). Whilst hydroxyapatite was similar on both SLA substrates, SLA250 stimulated the greatest amount of mineralization (Fig. 8). It has been reported that residual aluminum in high concentrations can inhibit matrix mineralization in osteoblasts derived from alveolar bone^39^ although no inhibitory affect from the SLA substrates was observed in the present study. ALP, which is accepted as an early stage marker of the differentiation process,^40^ was not affected by surface topography after 5 days in culture (Fig. 10).

The results of the present study is an early indication that surface modification of CoCrMo by sandblasting and acid etching may prove to be advantageous by enhancing cellular responses through increased levels of cell adhesion, proliferation, and osteogenic differentiation. It is highly likely that the combination of surface features on the micro‐ and nanoscale is influential to this result,^15^ as CoCrMo substrates with either nanophase and micron scale features have been shown to be superior over conventional CoCrMo.^41^, ^42^ In contrast, CoCrMo porous bead coated implants that underwent acid etching were shown to promote no significant improvement over control implants in a canine model^43^ emphasizing the sensitivity of topographical surface modification. This high degree of sensitivity was observed in the present study by the favorability of SLA250 over SLA50, when only a small difference in *R*_a_ of 0.2 µm existed between the two surfaces. It is likely that the combination of increased surface roughness and less aluminum contamination on SLA250 resulted in its superior performance.^26^, ^33^, ^39^ Further studies which focus on the removal of residual aluminum particles whilst maintaining an optimum level of surface roughness may prove to be advantageous for further enhancing the bioactivity of CoCrMo.

## CONCLUSION

This study assessed the behavior of human MSCs on four surface topographies created on CoCrMo. The results indicate the properties of the SLA250 surface promoted a superior level of bioactivity by way of enhancing cell adhesion, proliferation, and markers of osteogenic differentiation, despite the presence of low levels of aluminum residue. Application of this surface topography onto implants formed of CoCrMo may prove to be advantageous by enhancing bone formation which may result in reduced healing times and improved implant stability.

## References

[1] Murray CJL , Richards M , Newton J , Fenton K , Anderson HR , Atkinson C , Bennett D , Bernabé E , Blencowe H , Bourne R , Braithwaite T , Brayne C , Bruce N , Brugha T , Burney P , Dherani M , Dolk H , Edmond K , Ezzati M , Flaxman A , Fleming T , Freedman G , Gunnell D , Hay R , Hutchings S , Ohno S , Lozano R , Lyons R , Marcenes W , Naghavi M , Newton C , Pearce N , Pope D , Rushton L , Salomon J , Shibuya K , Vos T , Wang H , Williams H , Woolf A , Lopez A , Davis A. UK health performance: Findings of the global burden of disease study 2010. Lancet (London, England) 2013;381:997–1020. 10.1016/S0140-6736(13)60355-4 23668584

[2] Culliford DJ , Maskell J , Beard DJ , Murray DW , Price AJ , Arden NK . Temporal trends in hip and knee replacement in the united kingdom: 1991 to 2006. J Bone Joint Surg Br Volume 2010;92:130–135. 10.1302/0301-620X.92B1.22654 20044691

[3] Kurtz S , Ong K , Lau E , Widmer M , Maravic M , Gómez Barrena E , de Fátima de Pina M , Manno V , Torre M , Walter W , de Steiger R , Geesink RGT , Peltola M , Röder C. International survey of primary and revision total knee replacement. Int Orthop 2011;35:1783–1789. 21404023 10.1007/s00264-011-1235-5 PMC3224613

[4] Kurtz S , Ong K , Lau E , Mowat F , Halpern M . Projections of primary and revision hip and knee arthroplasty in the united states from 2005 to 2030. J Bone Joint Surg Am Volume 2007;89:780–785. 10.2106/JBJS.F.00222 17403800

[5] Kurtz S , Lau E , Ong K , Zhao K , Kelly M , Bozic K . Future young patient demand for primary and revision joint replacement: National projections from 2010 to 2030. Clin Orthop Rel Res 2009;467:2606–2612. 10.1007/s11999-009-0834-6 PMC2745453 19360453

[6] Puleo DA , Nanci A . Understanding and controlling the bone–implant interface. Biomaterials 1999; 20:2311–2321. 10614937 10.1016/s0142-9612(99)00160-x

[7] Logan N , Brett P . The control of mesenchymal stromal cell osteogenic differentiation through modified surfaces. Stem Cells Int 2013;2013:361637. 23766768 10.1155/2013/361637 PMC3674690

[8] Balloni S , Calvi E , Damiani F , Bistoni G , Calvitti M , Locci P , Becchetti E , Marinucci L . Effects of titanium surface roughness on mesenchymal stem cell commitment and differentiation signaling. Int J Oral Maxillofac Implants 2009;24:627–635. 19885402

[9] Zhang WJ , Li ZH , Liu Y , Ye DX , Li JH , Xu LY , Wei B , Zhang XL , Liu XY , Jiang XQ. Biofunctionalization of a titanium surface with a nano‐sawtooth structure regulates the behavior of rat bone marrow mesenchymal stem cells. Int J Nanomed 2012;7:4459–4472. 10.2147/IJN.S33575 PMC3422101 22927760

[10] Oh S , Brammer KS , Li YSJ , Teng D , Engler AJ , Chien S , Jin S . Stem cell fate dictated solely by altered nanotube dimension. Proc Natl Acad Sci USA 2009;106:2130–2135. 19179282 10.1073/pnas.0813200106 PMC2650120

[11] Lavenus S , Trichet V , Le Chevalier S , Hoornaert A , Louarn G , Layrolle P . Cell differentiation and osseointegration influenced by nanoscale anodized titanium surfaces. Nanomedicine 2012;7:967–980. 22394187 10.2217/nnm.11.181

[12] Sjöström T , Dalby MJ , Hart A , Tare R , Oreffo ROC , Su B . Fabrication of pillar‐like titania nanostructures on titanium and their interactions with human skeletal stem cells. Acta Biomater 2009;5:1433–1441. 19208503 10.1016/j.actbio.2009.01.007

[13] McNamara LE , Sjostrom T , Burgess KEV , Kim JJW , Liu E , Gordonov S , Moghe PV , Meek RMD , Oreffo ROC , Su B , Dalby MJ . Skeletal stem cell physiology on functionally distinct titania nanotopographies. Biomaterials 2011;32:7403–7410. 21820172 10.1016/j.biomaterials.2011.06.063

[14] Cochran D , Jackson J , Bernard J‐P , ten Bruggenkate C , Buser D , Taylor T , Weingart D , Schoolfield J , Jones A , Oates T . A 5‐year prospective multicenter study of early loaded titanium implants with a sandblasted and acid‐etched surface. Int J Oral Maxillofac Implants 2011;26:1324–1332. 22167440

[15] Mendonca G , Mendonca DBS , Aragao FJL , Cooper LF . The combination of micron and nanotopography by h2so4/h2o2 treatment and its effects on osteoblast‐specific gene expression of hMSCs. J Biomed Mater Res Part A 2010;94:169–179. 10.1002/jbm.a.32701 20128007

[16] Geetha M , Singh AK , Asokamani R , Gogia AK . Ti based biomaterials, the ultimate choice for orthopaedic implants—A review. Prog Mater Sci 2009;54:397–425.

[17] Logan N , Sherif A , Cross AJ , Collins SN , Traynor A , Bozec L , Parkin IP , Brett P . TiO2‐coated CoCrMo: Improving the osteogenic differentiation and adhesion of mesenchymal stem cells in vitro. J Biomed Mater Res Part A 2015;103:1208–1217. 10.1002/jbm.a.35264 25045159

[18] Tsaryk R , Peters K , Unger RE , Feldmann M , Hoffmann B , Heidenau F , Kirkpatrick CJ . Improving cytocompatibility of Co28Cr6Mo by TiO2 coating: Gene expression study in human endothelial cells. J R Soc Interface 2013;10:20130428. 23825117 10.1098/rsif.2013.0428 PMC3730696

[19] Logan N , Cross AJ , Traynor A , Bozec L , Parkin IP , Brett P . Mesenchymal stem cell response to UV‐photofunctionalized TiO2 coated CoCrMo. RSC Adv 2014;4:59847–59857.

[20] Att W , Hori N , Iwasa F , Yamada M , Ueno T , Ogawa T . The effect of UV‐photofunctionalization on the time‐related bioactivity of titanium and chromium‐cobalt alloys. Biomaterials 2009;30:4268–4276. 19473697 10.1016/j.biomaterials.2009.04.048

[21] Poh CK , Shi ZL , Tan XW , Liang ZC , Foo XM , Tan HC , Neoh KG , Wang W . Cobalt chromium alloy with immobilized BMP peptide for enhanced bone growth. J Orthop Res 2011;29:1424–1430. 21445991 10.1002/jor.21409

[22] Tan H , Poh C , Cai Y , Wang W . Anti‐fibrosis effect of BMP‐7 peptide functionalization on cobalt chromium alloy. J Orthop Res 2013;31:983–990. 23456668 10.1002/jor.22313

[23] Tan H , Poh C , Cai Y , Soe M , Wang W . Covalently grafted BMP‐7 peptide to reduce macrophage/monocyte activity: An in vitro study on cobalt chromium alloy. Biotechnol Bioeng 2013;110:969–979. 23055400 10.1002/bit.24756

[24] Durual S , Rieder P , Garavaglia G , Filieri A , Cattani Lorente M , Scherrer S . TiNOx coatings on roughened titanium and CoCr alloy accelerate early osseointegration of dental implants in minipigs. Bone 2013;52:230–237. 23000509 10.1016/j.bone.2012.09.014

[25] Dalby MJ , Gadegaard N , Tare R , Andar A , Riehle MO , Herzyk P , Wilkinson CDW , Oreffo ROC . The control of human mesenchymal cell differentiation using nanoscale symmetry and disorder. Nat Mater 2007;6:997–1003. 17891143 10.1038/nmat2013

[26] Wennerberg A , Albrektsson T . Effects of titanium surface topography on bone integration: A systematic review. Clin Oral Implants Res 2009;20:172–184. 19663964 10.1111/j.1600-0501.2009.01775.x

[27] Paredes V , Salvagni E , Rodriguez E , Gil FJ , Manero JM . Assessment and comparison of surface chemical composition and oxide layer modification upon two different activation methods on a CoCrMo alloy. J Mater Sci Mater Med 2014;25:311–320. 24202914 10.1007/s10856-013-5083-2

[28] Hayashi R , Ueno T , Migita S , Tsutsumi Y , Doi H , Ogawa T , Hanawa T , Wakabayashi N . Hydrocarbon deposition attenuates osteoblast activity on titanium. J Dent Res 2014;93:698–703. 24868012 10.1177/0022034514536578 PMC4293731

[29] Durual S , Pernet F , Rieder P , Mekki M , Cattani Lorente M . Titanium nitride oxide coating on rough titanium stimulates the proliferation of human primary osteoblasts. Clin Oral Implants Res 2011;22:552–559. 21087318 10.1111/j.1600-0501.2010.02033.x

[30] Wall I , Donos N , Carlqvist K , Jones F , Brett P . Modified titanium surfaces promote accelerated osteogenic differentiation of mesenchymal stromal cells in vitro. Bone 2009;45:17–26. 19332166 10.1016/j.bone.2009.03.662

[31] Rosales‐Leal JI , Rodriguez‐Valverde MA , Mazzaglia G , Ramon‐Torregrosa PJ , Diaz Rodriguez L , Rosales Leal JI , Rodríguez Valverde MA , Ramón Torregrosa PJ , Díaz Rodríguez L , García Martínez O , Vallecillo Capilla M , Ruiz C , Cabrerizo Vílchez MA . Effect of roughness, wettability and morphology of engineered titanium surfaces on osteoblast‐like cell adhesion. Colloids Surf A 2010;365:222–229.

[32] Aita H , Att W , Ueno T , Yamada M , Hori N , Iwasa F , Ogawa T . Ultraviolet light‐mediated photofunctionalization of titanium to promote human mesenchymal stem cell migration, attachment, proliferation and differentiation. Acta Biomater 2009;5:3247–3257. 19427421 10.1016/j.actbio.2009.04.022

[33] Sader M , Balduino A , Soares GA , Borojevic R . Effect of three distinct treatments of titanium surface on osteoblast attachment, proliferation, and differentiation. Clin Oral Implants Res 2005;16:667–675. 16307573 10.1111/j.1600-0501.2005.01135.x

[34] Khan MR , Donos N , Salih V , Brett PM . The enhanced modulation of key bone matrix components by modified titanium implant surfaces. Bone 2012;50:1–8. 21906701 10.1016/j.bone.2011.07.040

[35] Yamada M , Miyauchi T , Yamamoto A , Iwasa F , Takeuchi M , Anpo M , Sakurai K , Baba K , Ogawa T . Enhancement of adhesion strength and cellular stiffness of osteoblasts on mirror‐polished titanium surface by UV‐photofunctionalization. Acta Biomater 2010;6:4578–4588. 20633705 10.1016/j.actbio.2010.07.010

[36] Davies JE . Mechanisms of endosseous integration. Int J Prosthodont 1998;11:391–401. 9922731

[37] Davies JE . Understanding peri‐implant endosseous healing. J Dent Educ 2003;67:932–949. 12959168

[38] Le Guehennec L , Soueidan A , Layrolle P , Amouriq Y . Surface treatments of titanium dental implants for rapid osseointegration. Dent Mater 2007;23:844–854. 16904738 10.1016/j.dental.2006.06.025

[39] Canabarro A , Diniz MG , Paciornik S , Carvalho L , Sampaio EM , Beloti MM , Rosa AL , Fischer RG . High concentration of residual aluminum oxide on titanium surface inhibits extracellular matrix mineralization. J Biomed Mater Res Part A 2008;87:588–597. 10.1002/jbm.a.31810 18186053

[40] Golub EE , Boesze‐Battaglia K . The role of alkaline phosphatase in mineralization. Curr Opin Orthop 2007;18:444–448. 10.1097/BCO.0b013e3282630851.

[41] Webster TJ , Ejiofor JU . Increased osteoblast adhesion on nanophase metals: Ti, Ti6Al4V, and CoCrMo. Biomaterials 2004;25:4731–4739. 15120519 10.1016/j.biomaterials.2003.12.002

[42] Jaeger M , Urselmann F , Witte F , Zanger K , Li X , Jäger M , Ayers DC , Krauspe R . Osteoblast differentiation onto different biometals with an endoprosthetic surface topography in vitro. J Biomed Mater Res Part A 2008;86:61–75. 10.1002/jbm.a.31552 17941017

[43] Jakobsen SS , Baas J , Jakobsen T , Soballe K . Acid etching does not improve CoCrMo implant osseointegration in a canine implant model. Hip Int 2010;20:171–178. 20544657 10.1177/112070001002000207

